# Association study of angiotensin converting enzyme gene polymorphism with elderly diabetic hypertension and lipids levels

**DOI:** 10.1186/1476-511X-12-187

**Published:** 2013-12-19

**Authors:** Yun-Fei Zhou, Hui Yan, Xiao-Ping Hou, Jing-Li Miao, Jing Zhang, Qiao-Xiang Yin, Jun-Jie Li, Xiao-Yan Zhang, Yuan-Yuan Li, Hui-Lan Luo

**Affiliations:** 1 The cadre ward of General Hospital of the Air Force PLA, No. 30, Fucheng Road, Haidian District, Beijing 100142, China; 2 Combination of traditional Chinese and Western Medicine Hospital of Southern Medical University, Guangzhou 510515, China

**Keywords:** Angiotensin converting enzyme gene, Insertion/deletion polymorphism, Elderly, Hypertension, Diabetic hypertension

## Abstract

**Objective:**

To investigate the relationship between angiotensin converting enzyme (ACE) gene insertion/deletion (I/D) polymorphism and diabetic essential hypertension in elderly population.

**Methods:**

Polymerase chain reaction (PCR) technique was used in 260 elderly normal control patients, 205 elderly hypertensive patients and 138 elderly diabetic hypertensive patients to detect the I/D polymorphism in ACE gene.

**Results:**

DD genotype frequency (0.352) and D allele frequency (0.543) in elderly hypertensive patients were higher than those in the normal control patients. DD genotype (0.421) and D allele frequency (0.579) in elderly diabetic hypertensive patients were significantly higher than those in the control patients (0.133 and 0.250). The differences of DD genotype and D allele frequency between the elderly hypertensive patients and the elderly diabetic hypertensive patients were not significant (*P* > 0.05).

**Conclusion:**

ACE gene deletion is a risk factor for hypertension but is not a risk factor for diabetes in elderly population.

## Introduction

ACE is an important component of the renin - angiotensin system and plays an important role in hypertension and other cardiovascular and cerebrovascular diseases [1]. Type 2 diabetes is a polygenic complex disease caused by genetic factors and environmental factors [2], and the genetic factors are quite essential in the development of type 2 diabetes [3]. Many studies showed that the genetic polymorphisms of some genes related to metabolism were associated with type 2 diabetes [4], and the ACE gene polymorphism was one of them. There is a 287 bp DNA fragment insertion (insertion, I)/deletion (deletion, D) polymorphism in intron 16 of ACE gene [5], which was studied in-depth in more and more basic researches [6-10]. And the association between the ACE gene polymorphism and the pathology of hypertension or diabetes has been paid more attention recently [7-9,11-15]. The present study aimed to explore the association of ACE gene polymorphism with hypertensive patients and with diabetic hypertension patients in elderly population in China.

## Subjects and methods

### Subjects

260 subjects were enrolled in the elderly control group (Male, n = 150, Female, n = 110), whose age were from 75 to 85 years (81.6 ± 4.55 years). They were admitted to our hospital for medical examination and routine treatment from the October 2006 to March 2013. They did not have hypertension, diabetes, coronary heart disease or any other cardiovascular disease.

205 patients were enrolled in elderly hypertensive group (Male, n = 103, Female, n = 102), whose age were from 74 to 86 years (79.8 ± 6.28 years). Diagnosis of essential hypertension was defined according to the WHO diagnostic criteria of hypertension, namely as systolic blood pressure ≥ 140 mmHg, diastolic blood pressure ≥ 90 mmHg.

138 patients were enrolled in elderly diabetic hypertension group (Male, n = 70, Female, n = 68, whose age were from 75 to 83 years (79.12 ± 5.21 years). Diagnosis of diabetes is defined in accordance with the clinical criteria for diabetes.

## Methods

### Ethical statement

The present study has been performed with the approval of the ethics committee of General Hospital of the Air Force PLA and is in compliance with the Helsinki Declaration.

### Extraction of DNA template

The rapid DNA extraction kit (Jinan Xinbeixi Biotechnology Co., Ltd. Jinan, China) was used for the extraction of the template DNA according to the protocol of the Kit.

### PCR amplification of the target fragment

**Figure 1:**
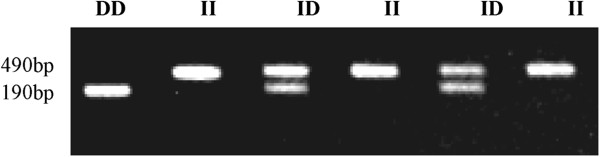
The genotyping results by 2.0% agarose.

Genotyping was conducted in accordance with kit instructions of the ACE gene I/D polymorphism assay (Jinan Xinbeixi Biotechnology Co., Ltd., Jinan, China). The primers sequences as follows: Upstream: 5’CCCAGGCCGGGGACTCTGTA-3’; Downstream:5’AGCTCCAGCCCTTAGCTCACCT3’. Genomic DNA, dNTPs and TaqDNA polymerase were used for PCR mixture and each primer were used for ACE. The PCR steps were as the following: initial denaturation step at 94°C for 4 minutes, followed by 36 cycles of denaturation at 94°C for 50 seconds, annealing at 58°C for 30 seconds, extension at 72°C for 60 seconds and a final extension at 72°C for 5 minutes. Amplified products had two fragments: fragment with length of 490 bp was defined I allele, fragment with length of 190 bp was defined D allele. II genotype had only 1 band of 490 bp, ID genotype had both 490 bp and 190 bp bands, DD genotype had 1 band of 190 bp (Figure 1).

### Statistical analysis

SPSS 13.0 software was utilized for the data analysis, The genotype frequencies and the allele frequencies of ACE gene among groups were compared using *χ*2 test, *P* <0.05 was considered significant difference.

## Results

### Hardy-Weinberg equilibrium test

ACE genotype distributions in three groups were in line with Hardy-Weinberg equilibrium (all *P* > 0.05, data not shown).

### Genotype distribution

Compared to the healthy control group, the frequency of ID and DD genotype was significantly higher in both elderly hypertensive patients group and in elderly hypertension with diabetes group (P <0.001). Similarly, the D allele frequency was also higher in these two groups comparing to the control group. We did not find significant significance between elderly hypertensive group and elderly hypertension with diabetes group in genotype or allele frequency (Table 1).

**Table 1:** ACE genotype and allele frequencies in elderly control group, elderly hypertensive group and elderly hypertensive and diabetic group

**Groups**	**n**	**Genotypes**			**Allele**	
		II	ID	DD	I	D
Control group	260	31 (11.9)	60 (23.1)	169 (65.0)	23.5	76.5
Hypertension group	205	61 (29.8)*#	78 (38.0)*#	66 (32.2)*#	48.8*#	51.2
Hypertension with diabetes group	138	44 (31.9)**	51 (37.0)**	43 (31.1)**	0.50**	0.50

Note: Between the elderly hypertensive group and the control group: *χ^2^ for genotypes = 20.09, P <0.001, χ^2^ for alleles = 23.63, P <0.001. Between the elderly hypertensive and diabetic group and the control group, **χ^2^ for genotypes = 10.62, P <0.001, χ^2^ for alleles = 22.4, P <0.001. Between the elderly hypertensive group and the elderly hypertensive and diabetic group, #χ^2^ for genotypes =0.68, P>0.05, χ^2^ for alleles = 0.29, P>0.05.

### Serum glucose and lipid levels in different genotypes

TG and LDL-C levels in patients with ID and DD genotype were significantly higher than those in patients with II genotype, the difference of blood glucose levels were not statistically significant in different genotypes (*P* > 0.05), Table 2.

**Table 2:** Lipids and glucose levels between each genotypes

**Genotypes**	**TG (mmol/L)**	**TC (mmol/L)**	**HDL-C (mmol/L)**	**LDL-C (mmol/L)**	**Glucose (mmol/L)**
II	1.4 ± 0.2	5.4 ± 1.1	0.9 ± 0.3	2.4 ± 1.1	4.6 ± 0.8
ID	1.8 ± 0.4*	6.3 ± 1.7*	1.2 ± 0.4*	3.1 ± 1.2*	5.7 ± 1.2*
DD	1.9 ± 0.3**	6.5 ± 1.4**	1.3 ± 0.3**	3.3 ± 1.3**	6.4 ± 1.3**

Note: Compared to II genotype: *P < 0.05, **P < 0.01.

## Discussion

Epidemiological studies show that the hypertension is a polygenic disease, and the genetic and environmental factors were involved in the pathogenesis of hypertension together. ACE is the key enzyme in renin - angiotensin system, which can catalyze the conversion of angiotensinI to angiotensin II and is the ACE protein coding gene [2,15]. ACE gene exists in chromosome 17q23 and contains 26 exons and 25 introns with a total length of 21 kb. Accordance with the presence or absence of 287 bp fragment in intron 16, there is an insertion/deletion (I/D) polymorphism. Some studies confirmed the presence of three genotypes: deletion homozygous genotype DD, insertion homozygous genotype II and heterozygous genotype ID.

Type 2 diabetes is a complex polygenic disease caused by genetic factors and environmental factors, of which, the genetic factors play the important role in the development of type 2 diabetes [3]. In recent years, the relationship between ACE gene polymorphism and type 2 diabetes was inconsistent among different reports. For example, in a recent published meta-analysis [16] report showed that a total of 41 studies including 4708 cases and 5368 controls were pool analyzed the association between ACE I/D polymorphism and T2DM in a Chinese population. The authors found the pooled ORs for the association between ACE I/D polymorphism and T2DM risk were not statistically significant under all genetic models (co-dominant model: DD vs. II: OR = 1.17, 95% CI 0.97-1.42 and ID vs. II: OR = 1.01, 95% CI 0.93-1.10; dominant model: OR = 1.06, 95% CI 0.94-1.19; multiplicative model: OR = 1.08, 95% CI 0.98-1.18). However, Zarouk et al. [17] reported that the DD genotype and the D allele are associated with hypertension and type 2 diabetes in Egyptian patients.

In the present study, we utilized the case - control study to analyze the relationship between the ACE gene polymorphism and elderly hypertension and diabetic hypertension. The role of ACE gene in essential hypertension remained controversial. Woo et al. [18] reported ACE gene was not observed to be associated with essential hypertension. However, in another population-based study, the D allele was found to be associated with hypertension [19]. In addition, Kenric et al. also found that the D allele was associated with hypertension in a group of African Americans [20].

In this study, between elderly hypertensive group, elderly hypertensive with diabetes group and healthy control group, the frequencies of ID and DD genotype in ACE gene, as well as the frequency of D allele, were significantly increased. Indicating that ACE gene deletion polymorphism was associated with the pathogenesis of hypertension in elderly. The present study also found that the ACE gene I/D polymorphism was not a risk factor for type 2 diabetes in the elderly population, which was consistent with some reports [21-23]. In addition, this study also found that in the different genotypes, TG and LDL-C levels in D allele carriers significantly increased, but it had no effect on blood glucose levels. The mechanism of ACE gene polymorphism affects the lipid levels was unclear, which is worth further exploring.

## Conclusion

In summary, in Chinese elderly population, DD genotype and D allele in ACE gene were associated with hypertension and lipid levels. However, they were not risk factors for type 2 diabetes in Chinese elderly population.

## Competing interests

The authors declared no competing interests exist.

## Authors’ contributions

YFZ and HLL carried out the molecular genetic studies and drafted the manuscript. HY and JZ carried out the genotyping. QX, JJL, and XYZ participated in the design of the study and performed the statistical analysis. YYL conceived of the study, and participated in its design and coordination and helped to draft the manuscript. All authors read and approved the final manuscript.
